# International web survey of chiropractic students about evidence-based practice: a pilot study

**DOI:** 10.1186/2045-709X-19-6

**Published:** 2011-03-03

**Authors:** Ryunosuke Banzai, Dustin C Derby, Cynthia R Long, Maria A Hondras

**Affiliations:** 1 Palmer Center for Chiropractic Research, Palmer College of Chiropractic, 741 Brady Street, Davenport, IA 52803-5209, USA; 2 Institutional Planning & Research, Palmer College of Chiropractic, 723 Brady Street, Davenport, IA 52803-5209, USA

## Abstract

**Background:**

Positive attitude toward evidence-based practice (EBP) principles in healthcare education may be one of the first steps for motivating a healthcare professional student to later apply EBP principles in clinical decision-making. The objectives for this project were to pilot an international web-based survey of chiropractic students and to describe student attitudes, behaviors, and knowledge about EBP principles.

**Methods:**

We used SurveyMonkey™ to develop our survey based on an existing questionnaire used to measure basic knowledge, skills and beliefs about EBP among allied healthcare professionals and CAM practitioners. We invited 26 chiropractic educational institutions teaching in English and accredited by official organizations to participate. Academic officials and registrars at participating institutions forwarded an invitation email and two reminders to students between July and September 2010. The invitation contained a link to the 38-item web-based questionnaire. Descriptive statistics were performed for analysis.

**Results:**

Fourteen institutions from Australia, Canada, US, Denmark and New Zealand participated. Among an estimated 7,142 student recipients of invitation letters, 674 participated in the survey for an estimated response rate of 9.4%. Most respondents reported having access to medical/healthcare literature through the internet, but only 11% read literature every week and 21% did not read literature at all. Respondents generally agreed that the use of research evidence in chiropractic was important. Although 76% of respondents found it easy to understand research evidence and 81% had some level of confidence assessing the general worth of research articles, 71% felt they needed more training in EBP to be able to apply evidence in chiropractic care. Respondents without previous training in research methods had lower confidence in assessing published papers. While more than 60% marked the correct answer for two knowledge items, the mean number of correct answers to the five knowledge questions was 1.3 (SD 0.9).

**Conclusions:**

Although it is feasible to conduct an international web survey of chiropractic students, significant stakeholder participation is important to improve response rates. Students had relatively positive attitudes toward EBP. However, participants felt they needed more training in EBP and based on the knowledge questions they may need further training about basic research concepts.

## Background

Since the early 1990's when the Evidence-Based Medicine Working Group at McMaster University[1] established explicit methodologies to determine "best evidence" for clinical medicine, many professional groups and organizations have emphasized the importance of evidence-based practice (EBP) for their practitioners[2-6]. The EBP movement emerged to facilitate clinical decision-making by healthcare professionals and their patients; however, both groups are challenged to know how, where, and when to seek out the best evidence to bridge gaps between research evidence and practical health outcomes[7].

Djulbegovic et al.[8] recently stated that "we should regard evidence-based medicine as a constantly evolving heuristic foundation for optimizing clinical practice, rather than a new scientific or philosophical theory that changes the nature of medicine." By virtue of the exponential growth of healthcare information of both high and low quality, acquisition of EBP principles requires certain knowledge and skills to synthesize the best available research evidence with other factors in clinical decision-making[9,10].

There are a number of ways available to deal with the difficulties and barriers of teaching EBP principles[11-18] and exposure to EBP principles in healthcare education has received considerable attention during the past decade[5,19-23]. The Accreditation Council for Graduate Medical Education[5] requires medical residents to demonstrate the ability to appraise and to integrate scientific evidence. Similarly, an understanding of the principles of EBP and the application of evidence into practice is part of the core training both of medical doctors and complementary and alternative medicine (CAM) healthcare practitioners in the United Kingdom[6]. Smith et al.[23] stated that the development of "evidence-based skills" should include early exposure and experience with fundamental literature searching and critical appraisal skills. Novice clinicians may then develop critical thinking skills and learn to apply those skills to clinical decision-making. Because scientific evidence application plays such a critical role in the clinical decision-making process, healthcare students should learn and implement best practices using EBP during their professional course of study. Chiropractic, one of the most widely used CAM disciplines,[24] is no exception.

Research on the inclusion of EBP principles in chiropractic curricula is lacking. Chiropractic students receive little formal instruction to generate searchable questions, conduct literature searches, critically appraise the literature or apply evidence to patient management[25,26]. Wyatt et al.[27] posited that instruction for EBP principles in chiropractic curricula in the US appeared to be deficient and an emphasis on "chiropractic philosophy" may promote unsupported beliefs and theories without research evidence. Other studies have found that chiropractic students have little interest in reading clinical research literature[23,28]. Understanding chiropractic student attitudes toward, knowledge of, and potential barriers and facilitators of using EBP principles may better inform curricular management and implementation of EBP at chiropractic educational institutions. Although several surveys of EBP have targeted health professionals, these data are lacking for health professional students, in general, and chiropractic students in particular.

The objectives of this study were to pilot a web-based survey of chiropractic students worldwide and to describe their attitudes, behaviors, and knowledge about EBP. Positive attitude toward EBP principles in healthcare education may be one of the first steps for motivating a healthcare professional student to later apply EBP principles in individual practice.

## Methods

We conducted an anonymous, cross-sectional, web-based international survey of chiropractic students. The Palmer College of Chiropractic Institutional Review Board approved this project.

### Eligibility Criteria

We set institutional and individual criteria for this project. Eligible institutions taught their curriculum in English and met criteria for accreditation from official organizations such as the Council on Chiropractic Education (CCE), the European CCE and CCE Australia. The CCE ensures the quality of chiropractic education by means of accreditation, educational improvement and public information, and requires institutions to teach research methods and procedures[29]. Of the 41 chiropractic educational institutions listed on the World Federation of Chiropractic quarterly report published on 30 September 2009,[30] 26 institutions met the eligibility criteria and were invited to participate in the study. Next, we invited students at least 18 years old who were enrolled in the chiropractic degree program at participating institutions. Students were not eligible if they were enrolled in bachelor programs other than chiropractic or in prerequisite studies to matriculate in a chiropractic program.

### Recruitment

We used both institutional and student recruitment strategies to reach our target population. First, we sent an invitation email to the Academic Deans or equivalent at the 26 eligible institutions asking for support of the project and permission to contact their registrar or their designee who maintains the chiropractic program student email distribution list. The institutional invitation described the purpose of the project, the secure and anonymous nature of data collection from students, and provided investigator contact information. We sent a second institutional invitation to non-responder institutions approximately three weeks later. Next, we prepared a similar communication to each institutional registrar of the institutions who granted permission and asked them to forward the student recruitment email invitation to all chiropractic students via their electronic distribution lists. Approximately three and five weeks later, we sent second and third invitation emails to the registrars to distribute to students.

### Survey Questionnaire

We used SurveyMonkey™to develop our survey based on the existing questionnaire that Hadley et al.[6] used to measure basic knowledge, skills and beliefs about EBP among allied healthcare professionals and CAM practitioners. First, we revised the instructions and items from the original questionnaire to target chiropractic students as opposed to practitioners. Second, we added five multiple choice knowledge questions that assessed fundamental critical appraisal concepts and four facilitator questions of interest with this student population. Third, our new questionnaire was a web-based version of the original paper questionnaire with the new items. Finally, we pre-tested and refined the questionnaire by inviting graduate clinical research fellows, research clinicians and clinical research project managers at our institutional research center to complete the survey; we incorporated feedback to improve clarity and readability of the instrument.

The final questionnaire had 38 items arranged in seven categories: attitudes, behaviors, facilitators, confidence, barriers, knowledge, and background information [see Additional file 1]. While the student respondents were anonymous, the invitation emails directed recipients to institution-specific Uniform Resource Locators (URLs) on SurveyMonkey™to track and estimate response rates by institution. The Consent to Participate page, the first screen of the questionnaire, included the title and purpose of the project, procedures for the anonymous and voluntary nature of the survey, and the potential risks and benefits to participants. Three questions followed the informed consent page to assess eligibility based on participant age, program of study, and whether or not they had already received a doctor of chiropractic or equivalent degree. Eligible participants were asked if they would like to participate in the survey with two choices; (1) No thank you, I decline to participate in this survey, and (2) Yes, I agree to participate in this survey. Choosing the second choice constituted participant consent to begin the survey. No questions other than those regarding eligibility required response. Despite a potential for duplication, we allowed multiple responses from the same computer to increase convenience for participants. There was neither password-protected access to the survey nor restriction for the range of the Internet Protocol (IP) address. The last page of the questionnaire thanked respondents for their participation and closed the window at the completion of the survey.

### Data Analysis

RB monitored the SurveyMonkey™web pages on a daily basis. RB downloaded data collected from survey participants from SurveyMonkey™, stored these data on the college's secure network, and transferred these data into SPSS statistical package version 17.0 (Chicago, IL) through Microsoft Office Excel^® ^for descriptive analysis. In addition to descriptive statistics for the recruitment process, we summarized the data as percentages and calculated means and standard deviations (SD) where appropriate.

## Results

**Figure 1:**
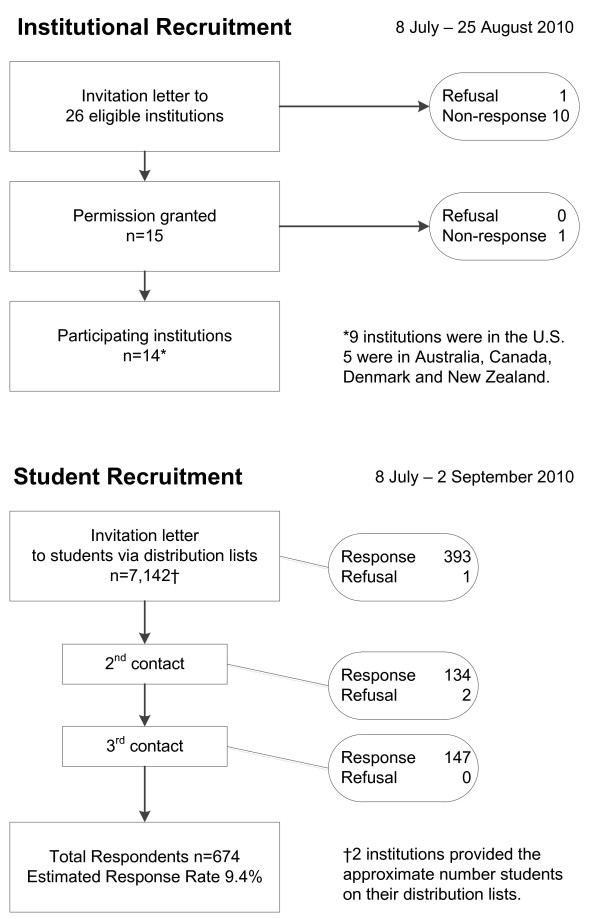
**Institutional and student recruitment efforts**.

Figure 1 displays the institutional and student recruitment efforts between July 8 and September 2, 2010. We sent invitation letters to the 26 eligible institutions on July 8 and 27, 2010. Fifteen institutions granted permission to contact their registrars and students. Of these, two institutions requested to review the ethics approval for the project before granting permission while 13 institutions granted permission based on our own institutional ethics approval. Among the 11 non-participating institutions, 10 did not respond to the institutional invitation while one refused due to perceived confidentiality concerns. We asked 15 registrars or their designee to forward the invitation letters to chiropractic students via their local distribution lists. One registrar did not respond. Of the 14 institutions who forwarded student invitations, nine institutions (87.5%) were from the US while the other five were from Australia, Canada, Denmark and New Zealand. Twelve institutions provided the exact number of students while the other two provided approximate numbers, for a total estimate of 7,142 students attending chiropractic programs at 14 institutions.

Of 740 persons who opened the link to the web survey, 674 were eligible and agreed to participate in the survey for an estimated 9.4% response rate. Three respondents declined to participate in the survey. Sixty three respondents were ineligible, some for multiple reasons: nine were younger than 18 years old, 32 were not students in a chiropractic program, and 38 had already received a doctor of chiropractic or equivalent degree. For the three waves of student recruitment, 393, 134 and 147 opened the survey link at the first, second and third contact, respectively. Students at Canadian Memorial College of Chiropractic received only one invitation because of summer recess during the recruitment process. Of 674 respondents, 171 (25.4%), 146 (21.7%) and 146 (21.7%) were from Life University, Palmer College of Chiropractic Davenport campus and New Zealand College of Chiropractic, respectively. Beyond the eligibility criteria, there were no additional required fields for the survey, so some items were incomplete.

The mean age of student respondents was 27.2 years (range 18-60). Table 1 displays additional respondent background information. While nearly three-fourths (72.5%) of respondents had no experience in the medical/healthcare field, two-thirds (66.6%) of respondents had taken at least one course about EBP or research methodology in their chiropractic education. There were 226 respondents (33.5%) who reported no formal education or training in research methods, epidemiology or statistics outside of the chiropractic curriculum, while 143 reported outside training in research methods, 52 in epidemiology, and 208 in statistics. Seventy-five students reported no EBP training either inside or outside of the chiropractic curriculum.

**Table 1:** Background information of respondents

Variable	Category	Percent
Gender (*n *= 476)	Female	48.9
	Male	51.1

Primary Language (*n *= 475)	English	88.4
	Others	11.6

What academic year are you in your chiropractic program? (*n *= 475)	Year1	25.7
	Year2	27.4
	Year3	26.7
	Year4	13.5
	Year5	6.7

Do you have any experience in the medical/healthcare field? (*n *= 477)	No	72.5
	Yes	27.5

Have you already taken a course related to evidence-based practice or research methodology in your chiropractic education? (*n *= 476)	None	33.4
	Once	46.4
	Twice	15.8
	> Twice	4.4

Have you been personally involved in conducting any kind of research? (*n *= 478)	None	58.8
	Once	27.4
	Twice	6.5
	> Twice	7.3

Among 571 respondents, 545 (95.6%) reported having access to medical/healthcare literature through the internet. There were 348/612 (56.9%) and 370/609 (60.8%) who searched for and read research evidence more than once a month, respectively. Only 69 of 611 respondents (11.3%) answered that they read every week regularly to keep up to date with medical/healthcare literature, while 229 (37.5%) read occasionally, 184 (30.1%) read only for specific information, and the remaining 129 (21.1%) answered that they do not keep up to date with medical/healthcare literature. Almost half of respondents (284/591, 48.1%) agreed that their institutions balanced philosophy, art and research evidence well.

Table 2 shows the mean (SD) and percentages of responses to each question about attitudes toward, facilitators, barriers and confidence of using EBP principles. Respondents had a mean of 4.8 (1 = Strongly Disagree, 6 = Strongly Agree) regarding the importance of the use of research evidence in chiropractic care and a mean of 4.2 regarding their ease in understanding research evidence. Almost all of the respondents (96%) reported being comfortable reading research evidence in English, and half of the respondents agreed that they had enough time to search and read research literature. Further, the mean confidence (1 = Not confident at all, 6 = Very confident) ranged from 3.6 in evaluating statistical tests in the open literature to 4.3 in assessing the general worth of research articles. However, the 75 respondents who had no previous training in research methods, epidemiology, statistics or EBP had lower confidence ranging from a mean of 3.2 in assessing study design to 3.8 in assessing the general worth of research articles.

**Table 2:** Attitudes, facilitators, barriers and confidence of using EBP

	Strongly Disagree	Disagree	Slightly Disagree	Slightly Agree	Agree	Strongly Agree	Mean*	SD
**Attitudes**								
1.I think that chiropractic is composed of a balanced combination between philosophy, art and science. (*n *= 636)	2.2	7.2	5.0	13.1	36.2	36.3	4.8	1.3
2. I think that the use of research evidence is an important factor in chiropractic care. (*n *= 636)	0.6	1.4	1.3	8.2	33.3	55.2	5.4	0.9
3. I think that research evidence has little impact on chiropractic care. (*n *= 631)	37.6	36.1	12.7	6.2	4.6	2.9	2.1	1.3
4. I think that evidence- based practice is a temporary fad. (*n *= 631)	38.4	35.8	13.2	6.8	3.6	1.9	2.1	1.2
5. I feel that I need more training in evidence- based practice to be able to apply research evidence into chiropractic care. (*n *= 635)	5.7	11.5	12.1	25.0	30.9	14.8	4.1	1.4
6. I find it easy to understand research evidence. (*n *= 635)	2.4	6.3	15.0	32.0	34.6	9.8	4.2	1.1

**Facilitators**								
1. I feel that my institution incorporates research evidence into chiropractic education well. (*n *= 592)	4.2	7.4	11.5	27.4	38.0	11.5	4.2	1.3
2. I have a good teacher(s) at my institution who is(are) familiar with evidence-based practice principles.(*n *= 591)	2.5	3.0	6.4	18.8	41.8	27.4	4.8	1.2
3. I have at least one good role model of chiropractor who is familiar with evidence-based practice principles.(*n *= 592)	1.7	6.6	7.1	16.0	37.3	31.3	4.7	1.2

**Barriers**								
1. I am comfortable reading research evidence in English. (*n *= 572)	2.8	0.2	1.0	7.0	32.5	56.5	5.4	1.0
2. I have enough time to search medical/healthcare literature. (*n *= 570)	9.8	21.4	19.5	28.8	17.2	3.3	3.3	1.3
3. I have enough time to read medical/healthcare literature. (*n *= 570)	8.4	20.5	23.0	30.2	15.6	2.3	3.3	1.3

	Not confident at all	Not very confident	Slightly not Confident	Slightly Confident	Confident	Very Confident	Mean†	SD
	
**Confidence**								
1. Assessing study design (*n *= 572)	7.2	12.5	11.6	36.6	26.5	5.6	3.8	1.3
2. Evaluating bias (*n *= 572)	4.7	7.4	10.4	31.8	37.1	8.6	4.2	1.2
3. Evaluating the adequacy of sample size (*n *= 570)	5.5	6.7	10.8	28.0	39.2	9.9	4.2	1.3
4. Assessing generalisability (*n *= 572)	5.4	8.6	14.8	30.4	34.3	6.5	4.0	1.3
5. Evaluating statistical tests/principles (*n *= 570)	7.4	14.1	18.2	33.2	22.2	4.9	3.6	1.3
6. Assessing the general worth of research articles (*n *= 570)	4.1	5.6	9.7	30.9	41.8	7.9	4.3	1.2

Numbers listed are percentages unless otherwise noted.

*Mean scores for Attitudes, Facilitators and Barriers: Likert scale where 1 = Strongly Disagree and 6 = Strongly Agree.

†Mean scores for Confidence: Likert scale where 1 = Not confident at all and 6 = Very confident.

Table 3 displays the results of the five items aimed to assess students' knowledge of fundamental research concepts. While more than 60% marked the correct answer for two knowledge items, less than half answered correctly for the other three items. In addition, approximately 30% of all participants elected not to answer any of the knowledge questions. The mean number of correct answers to the 5 knowledge questions was 1.3 (SD 0.9) for the 461 respondents who answered all 5 questions; only one individual answered all 5 questions correctly. Evaluating this by whether or not the respondent reported previous training in research methods, epidemiology, statistics or EBP showed that the mean number of correct responses was lowest for those who had no previous training (n = 69; mean 1.1; SD 0.8) and highest for those who reported two or more courses in their chiropractic program in addition to at least one course outside of their chiropractic program (n = 51; mean 1.5; SD 0.9).

**Table 3:** Knowledge questions with correct answers in bold

Questions	Answers	Respons
Which section of an article is the best section to evaluate when critical analysis of information is needed? (*n *= 479)	1. Abstract	17.1
	2. Introduction section	3.1
	**3. Methods section**	**48.0**
	4. Conclusions section	28.8
	5. References	2.9

Because three cases of a very rare brain cancer have been detected in children living in a small community located near a hazardous waste disposal site, local clinicians want to determine if they can identify risk factors associated with cancer development. They should conduct a _______ to address this question. (*n *= 474)	1. Case series	25.7
	2. Randomized clinical trial	7.4
	3. Prospective cohort study	22.6
	4. Cross-sectional study	16.9
	**5. Case-control study**	**27.4**

A randomized clinical trial is designed to compare two different treatment approaches for a disease/condition of interest. The purpose of randomization is to: (*n *= 470)	1. Obtain treatment groups of similar size	2.3
	2. Select a representative sample of patients for study	53.8
	3. Increase patient compliance with treatment	4.0
	**4. Obtain treatment groups with coparable baseline prognoses**	**37.4**
	5. Increase the prevalence of disease in both groups	2.3

A controversy occurred between the proponents of drug therapy and spinal manipulation for patients with asthma. To support their position, one party wrote, "Of 119 patients with asthma, 97 showed improvement following spinal manipulation." The inference that in patients with asthma, spinal manipulation is the therapy of choice is: (*n *= 470)	1. Correct	13.6
	2. Incorrect because the comparison is not based on rates	3.4
	**3. Incorrect because no control or comparison group is being used**	**60.9**
	4. Incorrect because no test of statistical significance is being made	16.6
	5. Incorrect because a cohort effect may be operating	5.5

The following spinal manipulation research was performed: 1,000 randomly selected children two years of age were given full spine manipulation once per month for 12 consecutive months, and then followed for 10 years. Of these, 80% were never afflicted with spine pain or spine related disease. Which is the most correct conclusion regarding the efficacy of spinal manipulation? (*n *= 466)	1. Spinal manipulation is an excellent preventive therapy because of the high rate of healthy children.	16.5
	**2. No conclusion is possible because no follow-up was made of children who did not receive spinal manipulation**.	**66.7**
	3. Spinal manipulation is not very effective because it should have produced a higher rate of healthy children.	1.7
	4. No conclusion is possible because no test of statistical significance was performed.	11.8
	5. The significant figure is 100% - 80% = 20%, the rate of acquiring spine pain or illness.	3.2

Numbers listed are percentages.

At the end of the questionnaire, participants had the opportunity to provide comments about the survey. Of 74 who entered comments, 12 liked the survey while six commented that the instructions and items in the questionnaire were unclear and confusing. Fifteen respondents suggested areas for survey improvement, such as adding a question to assess one's understanding of the definition of EBP and including more items related to background information. Another respondent stated that the use of the term 'doctor of chiropractic' may not be relevant in some regions.

## Discussion

The results of this pilot study indicate that while it is feasible to conduct a web-based international survey of students attending chiropractic educational institutions in a relatively short period of time, institutional and student recruitment will require significant effort to improve response rates. Despite the low estimated response rate (9.4%), the number of respondents was 674 which allowed us to describe student attitudes, behaviors and basic knowledge of EBP principles.

Hadley's survey[6] of allied healthcare professionals and CAM practitioners found that the majority felt that EBP was essential to their practice. Similarly, the majority of our chiropractic student respondents felt that use of research evidence is an important factor in chiropractic care (96.7%), that evidence-based practice is not a temporary fad (87.4%), that they need more training in EBP to apply the evidence in practice (70.7%), and had little to no exposure to research methods, epidemiology and statistics outside of the chiropractic curriculum (33.5%). Unlike Hadley's survey, our chiropractic student respondents had greater confidence in their ability to assess research study design, generalisability, evaluate bias, sample size and statistical tests. However, student responses to very basic critical appraisal concepts revealed low levels of knowledge that did not match confidence levels. In addition, nearly one-third of the survey respondents did not attempt to answer any of the knowledge questions.

Similar to other health professional training programs, EBP training must be included in chiropractic curricula to prepare future chiropractors to effectively practice evidence-based clinical decision-making. The minimum requirement listed in CCE standards (p. 18) states that doctor of chiropractic degree programs must include research methods and procedures, and "document how each subject appears in the curriculum and is integrated into a coherent degree program."[29] Historically, chiropractic college curricula met this criterion by including one 2-or 3-credit course in research methods or critical appraisal. Little has been published regarding the most effective strategies for EBP training in chiropractic educational institutions. Research of other health professional training programs shows that EBP concepts provided in a stand-alone course improve student knowledge but may not change student behavior and attitudes toward using evidence if it is not tied to clinical application[31]. Recent studies suggest that evidence-based clinical skills can be taught, but they are not necessarily continued into future practice[32,33]. Most educators advocate for a "whole-program approach" and clinically integrated teaching of EBP seeded throughout a curriculum is likely required for truly meaningful learning and application[34-37].

In the US, the National Center for Complementary and Alternative Medicine at the National Institutes of Health created a research education grant program to increase the quality and quantity of research content in institutions that train CAM physicians and practitioners. Four chiropractic educational institutions were awarded grants through this program in the past 5 years (PAR-040-97 and PAR-080996) and have worked toward integrating EBP concepts and practice into their curricula[38]. Evidence that EBP is now being weaved through coursework and extra-curricular activities at the awarded campuses is suggested in their 13 presentations and 1 workshop on these topics scheduled for the 2011 Association of Chiropractic Colleges-Research Agenda Conference[39].

Students' perceived competencies in EBP principles may overestimate the actual skill sets essential for clinical decision-making[19]. In our survey, students had a positive attitude about EBP, had some training in EBP or research methods in their chiropractic program, but did not demonstrate good knowledge in research evidence principles. Regardless of whether or not respondents reported previous training in research methods, epidemiology, statistics or EBP, the mean knowledge scores were very low. Given the positive attitude toward the value of EBP principles, the perceived need for additional EBP training, and the low level of knowledge demonstrated by respondents, it was surprising that half of respondents agreed they had enough time to search and read research literature. Future surveys with higher response rates may inform academic administrators at chiropractic educational institutions about the current perspectives of their students related to EBP principles and may inform decisions about implementing EBP principles in their curricula. Furthermore, they may capture shifts in the knowledge of students due to initiatives such as those in the US described above.

Several surveying recommendations flow from the current research. First, surveyors conducting research with students attending chiropractic institutions should gather baseline information about registrar recordkeeping. For instance, two institutions in the current study could not report accurate enrollment numbers for their institution. Second, researchers should negotiate access to student email lists. By doing so, they can more actively target survey solicitations and follow-up messages as well as assert better control for potential survey duplication. The use of student email lists also allows the researcher to accurately calculate response rates to the survey, as well as to better gauge respondent representativeness to the population post-data collection. If these recommendations are followed, it is likely that future efforts will require ethics approval at all institutions surveyed.

Future surveys of this population should consider questionnaire translation into other languages and use multiple recruitment methods. We only used one method of student recruitment, namely three waves of invitations from the institutional contacts who maintain student email distribution lists. Institution-specific and more creative student recruitment methods may increase the response rate because some students may not check college email regularly, but might respond to other recruitment methods.

### Limitations

Similar to other surveys, limitations of this study include survey error regarding coverage and non-response[40]. Being denied access to survey students within some of the chiropractic institutions, as well as simply not hearing back from others, means that the current study possessed a certain level of coverage error. The high levels of non-response for certain items, coupled with the overall low response rate for the survey, indicates a certain degree of response bias, i.e., those students who chose to respond to those items may significantly differ from those who omitted a response. Furthermore, the demographic items, including whether or not students had previous training or education in EBP or research methodology either within or outside the chiropractic curriculum, were positioned at the end of the survey following the knowledge items. If respondents closed their browser before answering the knowledge items, then these records included missing data for the demographic and training information, which prevented further assessments of response bias and generalisability. A final limitation of this study concerns the self-report nature of the data. Significant issues are associated with many types of self-report data, especially when the responses solicited by this instrument could be perceived as personal in nature, and in some cases could invite social criticism or social reprimand.

## Conclusion

The results of this survey indicate that although it appears feasible to conduct a web-based survey with chiropractic students, significant stakeholder participation is crucial to improve response rates. Students had relatively positive attitudes toward EBP. However, they felt they needed more training in EBP and based on the knowledge questions they may need further training about basic research concepts.

## Competing interests

The authors declare that they have no competing interests. None of the authors teach, employ, nor supervise students eligible for this project.

## Authors' contributions

RB conceived the idea for the survey. RB, MAH and DCD contributed to project design. RB and MAH prepared the invitation letters to institutions and students and replied to queries about the project. RB managed the data and prepared the first manuscript draft. RB and CRL analyzed the data. MAH, DCD, and CRL provided critical and constructive support for data analysis, interpretation and manuscript preparation. All authors edited and approved the final version of the manuscript.

## Supplementary Material

Additional file 1 **A copy of the questionnaire developed to measure chiropractic students' attitudes, behaviors and knowledge of EBP principles**. Click here for file
