# Supplementary material for: International web survey of chiropractic students about evidence-based practice: a pilot study
Source: Chiropr Man Therap. 2011 Mar 3;19:6. doi: 10.1186/2045-709X-19-6 (PMC3058064; doi:10.1186/2045-709X-19-6)
Supplement: Additional file 1 — A copy of the questionnaire developed to measure chiropractic students' attitudes, behaviors and knowledge of EBP principles. [file 2045-709X-19-6-S1.PDF]

Worldwide Survey of Chiropractic Students about Evidence-Based Practice

1. Informed Consent

Welcome to the Worldwide Survey of Chiropractic Students about Evidence-Based Practice!

We invite you to participate in a research study to learn more about evidence-based practice (EBP) principles from students attending chiropractic colleges around the world. This research is in partial fulfillment of the requirements for the Master of Science in Clinical Research at the Palmer Center for Chiropractic Research in Davenport, Iowa, USA. The Principal Investigator is Ryunosuke Banzai, B.App.Sc, B.C.Sc.

The purpose of this project is to describe attitudes toward, knowledge of, and potential barriers and facilitators for incorporating EBP principles in chiropractic curricula. This is the first large-scale effort to capture the voice of chiropractic students around the world who are enrolled in chiropractic colleges accredited by national or international councils.

Your participation will help shape the future of EBP teaching curricula. In addition to reading this form and submitting your consent to participate in this study, you are encouraged to print this page for your records. After providing consent, you will be asked to complete a survey about EBP principles as they relate to your chiropractic education. We anticipate that the survey will take 20 minutes to complete.

There are no anticipated risks to you as a result of participating in this survey. Any information you provide in this study will be anonymous, and all of the data will be analyzed and described in aggregate form only. Although there may be no direct benefit for your participation, the results of this study may inform academic officials at chiropractic institutions about the current status of EBP principles as perceived by chiropractic students.

Taking part in this research study is voluntary. You may choose not to take part at all. If you decide to participate in this study, you may stop participating at any time by simply closing your browser window. If you decide not to be in this study, or if you stop participating at any time, there will be no consequences of any kind. Participation or non-participation will not affect your student status or any other personal consideration or right you usually expect.

If you have any questions about the research study itself, you may contact the Principal Investigator or his Graduate Advisory Committee Chair.

Ryunosuke Banzai, B.App.Sc, B.C.Sc (Clinical Research Fellow)
Phone: +1.563.884.5282
Email: ryunosuke.banzai@palmer.edu

Maria Hondras, DC, MPH (Associate Professor and Graduate Advisory Committee Chair)
Phone: +1.563.884.5199
Email: maria.hondras@palmer.edu

Drs. Banzai and Hondras may be reached by post at:
Palmer Center for Chiropractic Research
741 Brady Street
Davenport, IA 52803-5209
USA

If you have questions, concerns, or complaints about your rights as a research participant, please contact:

Dana Lawrence, DC, MMedED (Human Protections Administrator)
Palmer College of Chiropractic
1000 Brady Street
Davenport, IA 52803-5209
USA
Phone: +1.563.884.5302
Email: dana.lawrence@palmer.edu

2. Informed Consent

1. Are you at least 18 years of age?

- ◯ No
- ◯ Yes

2. Are you a student in a doctor of chiropractic program? (not other programs such as Chiropractic Assistant, Chiropractic Technician, or Master's programs)

- ◯ No
- ◯ Yes

3. Have you received a Doctor of Chiropractic or equivalent degree?

- ◯ No
- ◯ Yes

3. Informed Consent

1. I understand that my participation in this study is voluntary and that I may refuse to take part at all or may stop participating at any time by closing the browser window. Would you like to participate in this survey?

- ◯ No thank you, I decline to participate in this survey
- ◯ Yes, thank you! I agree to participate in this survey

4. Attitude

1. Please read each statement carefully and then select the answer that reflects your views most closely.

	Strongly Disagree	Disagree	Slightly Disagree	Slightly Agree	Agree	Strongly Agree
I think that chiropractic is composed of a balanced combination between philosophy, art and science.	◯	◯	◯	◯	◯	◯
I think that the use of research evidence is an important factor in chiropractic care.	◯	◯	◯	◯	◯	◯
I think that research evidence has little impact on chiropractic care.	◯	◯	◯	◯	◯	◯
I think that evidence-based practice is a temporary fad.	◯	◯	◯	◯	◯	◯
I feel that I need more training in evidence-based practice to be able to apply research evidence into chiropractic care.	◯	◯	◯	◯	◯	◯
I find it easy to understand research evidence.	◯	◯	◯	◯	◯	◯

5. Collection and Dissemination of Evidence

Instructions:
Please read each statement carefully and then select the one best answer that reflects your views most closely, unless otherwise instructed.

1. How often, on an average, do you search for evidence?

- ◯ More than once a week
- ◯ Every 1-2 weeks
- ◯ Every 3-4 weeks
- ◯ Less than once a month
- ◯ Never

2. How often do you read research evidence?

- ◯ More than once a week
- ◯ Every 1-2 weeks
- ◯ Every 3-4 weeks
- ◯ Less than once a month
- ◯ Never

3. Do you keep up to date with medical/healthcare literature?

- ◯ Yes - read every week regularly
- ◯ Yes - read occasionally
- ◯ Yes - only for specific information
- ◯ No

4. What types of resources do you read to find information about evidence for your chiropractic education? ***(please mark 3 resources that you use)***

- [ ] Journals: review articles
- [ ] Journals: original research reports
- [ ] Textbooks
- [ ] Internet resources
- [ ] Hospital guidelines
- [ ] Hospital Intranet gudelines
- [ ] The Cochrane Library
- [ ] NICE guidelines (National Institute for Health and Clinical Excellence)
- [ ] Clinical Guidelines (BMJ publication)
- [ ] Evidence based medicine journal
- [ ] PubMed/MEDLINE
- [ ] EMBASE
- [ ] DynaMed
- [ ] ICL (Index of Chiropracic Library)
- [ ] Other (please specify)

6. Facilitators

Please read each statement carefully and then select the answer that reflects your views most closely.

1. I feel that my institution balances philosophy, art and evidence well.

- No, I feel that my institution focuses more on philosophy than on the other two
- No, I feel that my institution focuses more on art than on the other two
- No, I feel that my institution focuses more on evidence than on the other two
- Agree

2. I feel that my institution incorporates research evidence into chiropractic education well.

- Strongly Disagree
- Disagree
- Slightly Disagree
- Slightly Agree
- Agree
- Strongly Agree

3. I have a good teacher(s) at my institution who is(are) familiar with evidence-based practice principles.

- Strongly Disagree
- Disagree
- Slightly Disagree
- Slightly Agree
- Agree
- Strongly Agree

4. I have at least one good role model of chiropractor who is familiar with evidence-based practice principles.

- ◯ Strongly Disagree
- ◯ Disagree
- ◯ Slightly Disagree
- ◯ Slightly Agree
- ◯ Agree
- ◯ Strongly Agree

7. Confidence

1. Please select one response for each statement that best represents your level of confidence when assessing each of these aspects of a published paper.

	Not confident at all	Not very confident	Slightly not confident	Slightly confident	Confident	Very confident
Assessing study design	◯	◯	◯	◯	◯	◯
Evaluating bias	◯	◯	◯	◯	◯	◯
Evaluating the adequacy of sample size	◯	◯	◯	◯	◯	◯
Assessing generalisability	◯	◯	◯	◯	◯	◯
Evaluating statistical tests/principles	◯	◯	◯	◯	◯	◯
Assessing the general worth of research articles	◯	◯	◯	◯	◯	◯

8. Barriers

Instructions:
Please read each statement carefully and then select the one best answer that reflects your views most closely.

1. I am comfortable reading research evidence in English.

- ◯ Strongly Disagree
- ◯ Disagree
- ◯ Slightly Disagree
- ◯ Slightly Agree
- ◯ Agree
- ◯ Strongly Agree

2. Do you have access to medical/healthcare literature through the internet?

- ◯ No
- ◯ Yes

3. I have enough time to search medical/healthcare literature.

- ◯ Strongly Disagree
- ◯ Disagree
- ◯ Slightly Disagree
- ◯ Slightly Agree
- ◯ Agree
- ◯ Strongly Agree

4. I have enough time to read medical/healthcare literature.

- ◯ Strongly Disagree
- ◯ Disagree
- ◯ Slightly Disagree
- ◯ Slightly Agree
- ◯ Agree
- ◯ Strongly Agree

9. Knowledge

Instructions:
Please read each statement carefully and then select the one best answer.

1. Which section of an article is the best section to evaluate when critical analysis of information is needed?

- Abstract
- Introduction section
- Methods section
- Conclusions section
- References

2. Because three cases of a very rare brain cancer have been detected in children living in a small community located near a hazardous waste disposal site, local clinicians want to determine if they can identify risk factors associated with cancer development. They should conduct a _______ to address this question.

- Case series
- Randomized clinical trial
- Prospective cohort study
- Cross-sectional study
- Case-control study

3. A randomized clinical trial is designed to compare two different treatment approaches for a disease/condition of interest. The purpose of randomization is to:

- Obtain treatment groups of similar size
- Select a representative sample of patients for study
- Increase patient compliance with treatment
- Obtain treatment groups with comparable baseline prognoses
- Increase the prevalence of disease in both groups

4. A controversy occurred between the proponents of drug therapy and spinal manipulation for patients with asthma. To support their position, one party wrote, "Of 119 patients with asthma, 97 showed improvement following spinal manipulation." The inference that in patients with asthma, spinal manipulation is the therapy of choice is:

- ◯ Correct
- ◯ Incorrect because the comparison is not based on rates
- ◯ Incorrect because no control or comparison group is being used
- ◯ Incorrect because no test of statistical significance is being made
- ◯ Incorrect because a cohort effect may be operating

5. The following spinal manipulation research was performed: 1,000 randomly selected children two years of age were given full spine manipulation once per month for 12 consecutive months, and then followed for 10 years. Of these, 80% were never afflicted with spine pain or spine related disease. Which is the most correct conclusion regarding the efficacy of spinal manipulation?

- ◯ Spinal manipulation is an excellent preventive therapy because of the high rate of healthy children.
- ◯ No conclusion is possible because no follow-up was made of children who did not receive spinal manipulation.
- ◯ Spinal manipulation is not very effective because it should have produced a higher rate of healthy children.
- ◯ No conclusion is possible because no test of statistical significance was performed.
- ◯ The significant figure is 100% - 80% = 20%, the rate of acquiring spine pain or illness.

10. Background

Instructions:
Please answer each question.

1. Your age

2. Gender

- Female
- Male

3. What is your primary language?

4. What academic year are you in your chiropractic program?

- Year 1
- Year 2
- Year 3
- Year 4
- Year 5

5. Do you have any experience in the medical/healthcare field?

- No
- Yes

If Yes, please specify.

6. Have you already taken a course related to evidence-based practice or research methodology in your chiropractic education?

- None
- Once
- Twice
- Three or more times

7. Outside of the chiropractic curriculum, have you had any formal education or training (i.e., degree course, seminar, workshop) in any of the following? *(mark all that apply)*

- ☐ None
- ☐ Research methods
- ☐ Epidemiology
- ☐ Statistics

8. Have you been personally involved in conducting any kind of research?

- ○ None
- ○ Once
- ○ Twice
- ○ Three or more times

9. We would like to hear how we can improve this survey, and please provide us with any additional comments you may have.

11. Thank you

Thank you for opening the link and reading the informed consent. Although your interest and responses are very valuable, we are afraid that you are not eligible for this survey. Please click the "Next" button below, and this will direct you to the last page of the survey. If you have any questions, please contact Dr. Ryunosuke Banzai, the Principal Investigator of this project at +1.563.884.5282 or ryunosuke.banzai@palmer.edu.

Again, we sincerely appreciate your interest in this survey.

Worldwide Survey of Chiropractic Students about Evidence-Based Practice

12. Thank you

Thank you for opening the link, and reading the informed consent page. While we are sorry that you decided not to participate in this survey, we also respect your decision.

1. Will you please describe the reason for declining to participate in this survey?

Please click the "Next" button below, and this will direct you to the last page of this survey. If you have any questions, please ask Dr. Ryunosuke Banzai, the Principal Investigator of the project at +1.563.884.5282 or ryunosuke.banzai@palmer.edu.

13. Thank You

Thank you very much for taking time for this survey! Your responses are very important to us and will remain anonymous contributions for this research study.
